# Supplementary material for: A local community on a global collective intelligence platform: A case study of individual preferences and collective bias in ecological citizen science
Source: PLoS One. 2024 Aug 26;19(8):e0308552. doi: 10.1371/journal.pone.0308552 (PMC11346665; doi:10.1371/journal.pone.0308552)
Supplement: S1 Appendix — (DOCX) [file pone.0308552.s001.docx]

A local community on a global collective intelligence platform: A case study of individual preferences and collective bias in ecological citizen science

# Appendix A: Questionnaire

This is a translation of the original questionnaire that was sent to the study’s participants:

**A study of Tatzpiteva**

Dear Tatzpiteva Participant,

As part of a study evaluating the Tatzpiteva project, we wish to learn about the monitoring patterns of the volunteers participating in the Tatzpiteva project. To this end, we would appreciate your cooperation in completing the enclosed questionnaire. The time required to complete the questionnaire is between 5 and 10 minutes.

At the end of the questionnaire, you are asked to provide your name (or Tatzpiteva username), which will enable us to correlate the patterns of monitoring with the data that emerges from the completed questionnaires. Please note that no other personal information is needed and the names will be deleted after the analysis is concluded. Once results are available, they will be shared with the Tatzpiteva community.

Many thanks in advance for your cooperation,

Author 1

University affiliation

Email address

*You are welcome to address any questions you may have to the Author.

===================================================================

**Questionnaire**

Part 1. Questions

1. Your reports to Tatzpiteva are based on time spent collecting field observations. Please indicate what percent of your total activity is dedicated to each time slot described below.

- Less than 1 hour: ________% of activity
- 1-2 hours: ________% of activity
- 2-4 hours: ________% of activity
- More than 4 hours: ________% of activity
- Random (rather than planned observations): ________% of activity

1. Please rank the animals observed by Tatzpiteva participants to reflect your preferences or the strength of your or emotional connection to each (1=most preferred; 9= least preferred; the list is alphabetical: please draw a line between the item and your ranking of it.)

| Fox | 1 |
| --- | --- |
| Gazelle | 2 |
| Hedgehog | 3 |
| Jackal | 4 |
| Mole | 5 |
| Mongoose | 6 |
| Porcupine | 7 |
| Tortoise | 8 |
| Wild Boar | 9 |

1. How do you decide which observations to report and which to omit? What are the criteria you consider? (For example, species uniqueness; its color, size, or symbolic value; a past personal experience)

__________________________________________________________________________________________________________________________________________________________________________________

1. Are your observations oriented towards a particular species? If yes, which one?

_______________________________________________________________________

Do you go out into nature with the specific purpose of monitoring and reporting on this/these species?

_______________________________________________________________________

- 1. During your monitoring activities, how likely were you to have observed a **tortoise** yet refrained from reporting this observation?
- Zero probability
- Low probability
- Medium probability
- High probability
- Almost certainly

What was your reason for not reporting this observation?

__________________________________________________________________________________________________________________________________________________________________________________

- 1. During your monitoring activities, how likely were you to have observed a **jackal** yet refrained from reporting this observation?
- Zero probability
- Low probability
- Medium probability
- High probability
- Almost certainly

What was your reason for not reporting this observation?

______________________________________________________________________________________________________________________________________________

- 1. During your monitoring activities, how likely were you to have observed a **gazelle** yet refrained from reporting this observation?
- Zero probability
- Low probability
- Medium probability
- High probability
- Almost certainly

What was your reason for not reporting this observation?

______________________________________________________________________________________________________________________________________________

- 1. During your monitoring activities, how likely were you to have observed a **wild boar** yet refrained from reporting this observation?
- Zero probability
- Low probability
- Medium probability
- High probability
- Almost certainly

What was your reason for not reporting this observation?

______________________________________________________________________________________________________________________________________________

**Thank you very much for your cooperation!**
