# Supplementary material for: A local community on a global collective intelligence platform: A case study of individual preferences and collective bias in ecological citizen science
Source: PLoS One. 2024 Aug 26;19(8):e0308552. doi: 10.1371/journal.pone.0308552 (PMC11346665; doi:10.1371/journal.pone.0308552)
Supplement: S2 Appendix — (DOCX) [file pone.0308552.s002.docx]

A local community on a global collective intelligence platform: A case study of individual preferences and collective bias in ecological citizen science

# Appendix B: Interview Protocol

Guidelines for semistructured interviews:

1. Demographic info, occupation
2. Reason for joining Tatzpiteva? What were you looking to gain?
3. How frequently do you participate?
4. What kind of Flora would you document/photograph? Any kind? Regardless of whether you can identify it?
5. What characterizes your decision to photograph an item?
6. When do you tend to monitor, weekdays? Weekends?
7. What would you say you have gained by participating? Please feel free to mention any aspect, emotional social, informational, practical, etc.
8. Do you participate in the group’s organized frameworks? The WhatsApp group? Group surveillance hikes?
9. How do you decide what to photograph?
10. What equipment do you use to take photos?
11. How do you decide whether to upload something onto the site?
12. How do you upload to the Website? During the observation or after? From the smartphone or directly from the online site?
13. What else do you use the website for?
14. What is your opinion regarding the quality of information posted on the Website?
15. Do you have any suggestions for technological improvements?
16. What would lead/enable you to increase your participation?
