# Supplementary material for: A local community on a global collective intelligence platform: A case study of individual preferences and collective bias in ecological citizen science
Source: PLoS One. 2024 Aug 26;19(8):e0308552. doi: 10.1371/journal.pone.0308552 (PMC11346665; doi:10.1371/journal.pone.0308552)
Supplement: S3 Appendix — (DOCX) [file pone.0308552.s003.docx]

A local community on a global collective intelligence platform: A case study of individual preferences and collective bias in ecological citizen science

# Appendix C: Code Book

| **Code** | **Description** |
| --- | --- |
| **Recordability** | **The ability to record a species once it has been detected** |
| Photographing equipment enabling or inhibiting the ability to record | The camera that is used by the observer enables/inhibits recording the species at the specific time and location (at a satisfactory resolution) |
| Danger prevents recording | Danger prevents the ability to record the species at the specific time and location |
| **Convenience** | **Choosing not to record or report because of the extra effort and time that are required** |
| Convenience related to recording decisions | Considerations regarding what [not] to *record* due to the effort and time that are required (with reference to specific species, locations or times) |
| Convenience related to reporting decisions | Considerations regarding what [not] to *report* due to the effort and time that are required |
| **Community value** | **Choosing to record and report because of the observation's importance to the project's goals** |
| The ability to identify species | The community's ability to identify the photographed species |
| Species' rarity or abundance | Choosing to record a species that is rare, or selecting not to record an abundant species |
| Importance to the community | Choosing to record an observation that is important for other members of the local community or to other viewers of the data |
| Importance to archive of observations | It is important to document observation and to create an archive of the region's biodiversity |
| General importance-related considerations | Either general statements related to importance (e.g. "everything is important") or ambiguous importance-related statements |
| **Personal Considerations** | **Personal considerations that influence the decision regarding what, where and when to record** |
| Personal preference for a particular species, region or time | Choosing to report a species, place or time because of a personal preference or attachment |
| Noteworthy features | Choosing to report an observation with unordinary features (e.g. color, size) or beauty |
| Learning from feedback | Recording and reporting an observation with the intent to learn its identity |
| Limiting one's repeated observations | Preferring not to repeat one's observations for species that s/he regularly reports |
| General personal choice (unexplained) | Indication of a choice for particular species, region or time, without an explanation |
