# Supplementary material for: A local community on a global collective intelligence platform: A case study of individual preferences and collective bias in ecological citizen science
Source: PLoS One. 2024 Aug 26;19(8):e0308552. doi: 10.1371/journal.pone.0308552 (PMC11346665; doi:10.1371/journal.pone.0308552)
Supplement: Attachment — Submitted filename: CitizenScienceBiases - PLOS ONE - R&R1 - RebuttalLetter.docx [file pone.0308552.s005.docx]

**PONE-D-23-35664: A local community on a global collective intelligence platform: A case study of individual preferences and collective bias in ecological citizen science**

**Rebuttal Letter**

Dear Prof. Hong Qin, Academic Editor, *PLOS One,*

We would like to thank the Academic Editor and reviewers for their valuable feedback and their insightful comments. We have worked hard to address the important concerns raised by the review team. As a result, the paper has improved along four key dimensions:

- **Motivation and framing:** We now better motivate the need to study individual observers’ considerations, and the way in which these aggregate to biases in the community-generated archive. Namely, on one hand we review prior works that discuss the risks to biased in CS ecological monitoring; on the other hand, we review recent papers that demonstrate that CS data is being employed for addressing some ecological questions. The motivation for this study is, then, the need to understand when biases pose a serious threat to researchers’ ability to employ the data and when biases are less of a concern.
- **Literature review:** Addressing the reviewers’ comments required that we go back to the literature. We leveraged this opportunity and performed an up-to-date review of relevant prior works. As a result, the manuscript added detail and many of the arguments are now better supported.
- **Paper organization and quality of writing:** We recruited the help of a professional copywriter, and together worked hard to address reviewers’ comments re writing quality, improve the flow of arguments, and articulate a clear narrative. As a results, some parts of the paper were moved, others deleted, and new paragraphs were introduced. Furthermore, the paper’s structure has also changed, adding new subsections. We believe that the revised manuscript reads much better now.
- **Details of study’s method.** The revised manuscript also includes 3 new appendices - (a) Questionnaire; (b) Interview protocol; and (c) Codebook for thematic analysis - adding important information about the study’s method.

Below, we expand on these issues and respond to each of the editor’s and reviewers’ comments point-by-point.

In addition to the rebuttal letter and revised manuscript, we have also submitted a file that tracks the changes we have made to the manuscript.

We thank the review team for helping us to improve the paper, and appreciate their constructive comments.

We believe the current manuscript is stronger both theoretically and empirically, and hope that our paper is now ready for publication at *PLOS One*.

**Academic Editor (AE)**

**AE.0** Thank you for submitting your manuscript to PLOS ONE. After careful consideration, we feel that it has merit but does not fully meet PLOS ONE’s publication criteria as it currently stands. Therefore, we invite you to submit a revised version of the manuscript that addresses the points raised during the review process.

Response: Thank you for affording us the opportunity to revise and resubmit the paper.

**Reviewer 1 (R1)**

**R1.0** Dear colleagues, thank you very much for the possibility of reviewing this manuscript about potential biases induced by individual and personal attitudes and preferences in an ecological citizen science project. I enjoyed reading the paper and I find the subject very topical for many aspects, from the mere evaluation of citizen science approaches to the indirect relevance of directly involving people in monitoring environmental changes.

Response: We would like to thank Reviewer 1 for the positive input. It is much appreciated!

**R1.1** In general I find the paper well written and the supporting literature exhaustively mentioned. However, I also find the text flow sometimes unnecessarily redundant (similar considerations in the introduction and in the discussion e.g.) and to some extent confusing, as far as the captions are concerned: for instance at page 4 (apparently the end of the introduction) where there is a hint to the results ("Our results reveal four primary factors that ...") without having even mentioned the research questions and/or the methodological approach (at this point there is a caption called 'Background', which should be mentioned earlier, maybe). Please check again the sequence and contents of the single captions. This will help the reader to follow the study.

Response: We agree with R1 that the paper flow was not ideal. Hence, we revised the structure and the writing. We changed and added several subsections, to better guide the reader. For example, in the Methods section we added a subsection describing the participants, and another section describing the data sources, which is also divided into 3 subsections with subtitles. We also stated our 2 research questions more clearly, and divided the results section accordingly: "The primary factors underlying participants’ reports" (RQ1) and "Amassing individuals’ preferences – biases in the aggregate database" (RQ2).

**R1.2** My major concerns regard the method: the paper reads that "roughly 40,000 observations were reported on Tatzpiteva by 400 observers, making up roughly half of all the iNaturalist observations in Israel. Most of Tatzpiteva’s observations were contributed by the community’s core members, where most members are peripheral and contribute only occasionally." Would it be possible to be more precise about the so called core members (how many are they and how many observations did they deliver?) as well as about the so called peripheral members? Are the core members the 38 persons that were interviewed?

Response: We added a new subsection in the Methods chapter, describing the participants in our study. In this new section, we clarified the distinction between peripheral and core members, and explain that we have initially identified 38 members as “core” members of the community (Crowston et al., 2006), based on multiple criteria. As we further explain in this new subsection, we have sent our questionnaire to all 38 members identified as core members of the community. Of those, 27 have answered our questionnaire. We then checked and saw that these 27 participants were responsible for 82% of the recorded observations in the entire Tatzpiteva project. Eight of them had been formally assigned “curator privileges” in the Tatzpiteva project, a position that corresponds to an administrator status in other online communities.

In the next stage, 15 of the questionnaire responders were interviewed, six of whom held curator responsibilities. So the bottom line is that we have gotten responses from the 27 most active, most committed members of the community, who together are responsible for over 80% of the activity on the platform; and interviewed a little more than half of them (including 75% of the project’s curators).

**R1.3** It would be appreciated if the entire questionnaire would be made available as a supplementary material (e.g.) instead of listing the questions in the text flow.

Response: We followed the Reviewer's recommendation and included the questionnaire in Appendix A. In the manuscript’s text, we emphasize the goals of the questionnaire and refer only to the open-ended questions.

**R1.4** Regarding the questionnaire, it is not fully clear to me, why only one species was chosen as highly likely to be observed whereas three species were listed as highly unlikely -why not having two likely and two unlikely species?

Response: In the questionnaire, in order to ground the questions regarding observers’ reporting behavior, we asked participants to focus on a limited number of species that are common in the region and thus participants have likely encountered them. To sample a broad range of preferences, constraints and behavior, we have selected species that vary in terms of their: *detectability* (determined by animal size, skin pattern and camouflage), *recordability* (which depends heavily on the speed in which animals move), and *rarity*.

We explain this in the revised Methods section

**R1.5** Table 1 show a very broaden distribution of the age and the contributions (number of reported observations) of the 27 participants: I am wondering (i) if it would be possible to add a hint to the standard error (or standard deviation) for both these measurements and most important (ii) how representative the sample is (27 out of 400 observers in sum, this is less than 10%; a maximum of approx. 3,500 reported observation out of a sum of 40,000, the same here). I would appreciate reading some considerations about this in the discussion.

Response: We have now added details of standard deviation to the table; indeed, there is much variation in members’ activity. As we mention in our response to R1.2 above, the newly added “Participants” subsection (under “Materials and Methods”) now provides a clear explanation of who our participants were, how they were selected, and why their contributions and considerations are, in fact, representative of the community’s output. As we explain, collectively, the 27 members who completed our questionnaire, contributed more than 80% of the observations in this project.

**R1.6** I am not a social scientist and I am not confident with citing answers from questionnaires in a paper, so while begging for understanding for my ignorance, I wonder (again) to which extent citing single statements of a low number of participants is representative for the study topic.

Response: Using exemplary quotations of study subjects in order to illustrate their way(s) of thinking is a staple of reporting field work in the social sciences, such as when writing ethnographies and case studies. This approach is a common practice, especially when taking a grounded theory or similar methodological approaches, such as thematic analysis (as we did). Using exemplary quotations of study applies whether quotations are heard (e.g. during interviews or observations), or collected in written form (form questionnaires, archives, etc.). This approach is presented, advocated, and touted in many textbooks about qualitative research (e.g. Bernard, 2017; Charmaz, 2006; Emerson et al., 2011), and widely taught in graduate programs in social sciences. As Bernard (2017, p. 468) explains:

*“[…] one of the most important methods in text analysis is the presentation of direct quotes from respondents—quotes that lead the reader to understand quickly what it took you months or years to figure out. You choose segments of text—verbatim quotes from respondents—as exemplars of concepts and theories”*.

This, enables

*“[…]to bring your reader close to your research participants and to the real-world situations described in your report.”.* Johnson and Christensen (2016).

Given R1’s comments, and the multidisciplinary nature of PLOS ONE and the diversity of its readers, we realized that many potential readers of this paper may be familiar with this genre / practice; therefore we added a short explanation about this at the beginning of the “Results” section, with appropriate references to relevant literature.

**R1.7** In the discussion I would appreciate some considerations for possible biases in studies on similar topics conducted by scientists instead of interested volunteers, for instance, and how combining the two approaches could maybe generate a win-win situation for everybody.

Response: Indeed, traditional scientific approaches for biodiversity monitoring, too, suffer from biases. They are prone to spatial biases (e.g. biodiversity research covers developed countries much more that the global south), temporal biases (most reports done during daytime, so diurnal animals investigated more than nocturnal animals) and taxonomic biases (e.g. shallow waters fish investigated more when compared to deep-waters fish; large mammals researched more than many of the micro-organisms). Still, scientific monitoring protocols are more controlled, and consequently less prone to biases when compared to data from unsystematic monitoring.

Recently, researchers have attempted to combine data from systematic and unsystematic (i.e. citizen science) sources. Preliminary attempts in this direction suggest that CS data could complement data from systematic monitoring protocols in the assessment of the state of nature (Comay et al., 2020).

The revised “Discussion” section briefly touches on this issue.

**R1.8** Please check the references once more, some of them did not get a number and are included in the text flow (see for instance Hochmair et al., 2020 at page 2 and Arazy & Malkinson, 2021 (page 27) in the discussion.

Response: We reviewed all of the references and numbered each to ensure that all are included in the list. (Note that in this revision, we introduced many new references).

**R1.9** Please carefully check the language again - sometimes I had the feeling that the sentences were incomplete (but I am not a native speaker, so I might be biased). E.g. page 2: "In particular, and the local organization of these communities could possibly hinder their ability to produce unbiased outcomes."

Response: We have revised the manuscript thoroughly, re-organized its flow, introduced new subtitles, added references, etc. We have also recruited the services of a professional copyeditor, who assisted us in this process. We believe that all these issues have now been alleviated.

**Reviewer 2 (R2)**

**R2.0** I read this manuscript with great interest. The authors report qualitatively on a multi-year citizen science project to collect data about the flora and fauna of Israel. The key focus of this manuscript is on biases in data arising from characteristics of contributors in deciding which observations to report. Overall, this manuscript provides useful qualitative insights into "what makes citizen scientists "tick" in the context of a specific project. The four characteristics of recordability, collective considerations, personal preferences, and convenience are intuitive and useful. The detailed comments from contributors help contextualize the findings. I do have several concerns and suggestions for improvement:

Response: We would like to thank Reviewer 2 for the constructive feedback, as well as for pointing out some strong aspects of the paper. We do appreciate this.

**R2.1** There seems to be an underlying assumption that collectively, contributions will "cancel out" biases of individual contributors. However, I do not see the validity of using this as a starting point. Geographic biases (e.g., concentrations near towns, roads, etc.) will not be overcome necessarily by having more contributors, nor will temporal biases, or biases toward reporting rare species or species that are hard to locate (e.g., nocturnal). These limitations seem built in to many CS projects.

Response: We thank you for this comment, which made us think hard about the paper’s motivation and framing.

Indeed, as the reviewer suggests, such biases may be built-in to many CS projects, and it may therefore seem “obvious” that ecological CS projects would suffer from spatial, temporal and taxonomic biases (as we review under the section “Challenges associated with CS data”).

However, a review of the relevant literature reveals a more nuanced reality. Importantly, not of individuals’ personal preferences and dispositions will end up translating to a collective bias in the aggregate database of observations; and, crucially: not all biases pose a real concern to researcher’s ability to answer ecological questions. For example, as we explain in the revised manuscript, some ecological research questions, e.g. those pertaining to species’ distribution and richness, are susceptible to those biases. The revised manuscript explains this in detail. An excerpt reads:

“The ability to use citizen-generated data (in particular, from unstructured monitoring) hinges on the quality of these data. Data quality is often defined in terms of its “fitness for use” (Eppler, 2006; Wang & Strong, 1996), a definition that is commonly applied to geographic information (Juran et al., 1979; Veregin, 1999). In order to assess the “fitness for use” of citizen-generated biodiversity data, we must consider the possible uses of these data. Recent research has demonstrated that CS data (including data from sporadic monitoring) can be valuable for addressing a variety of research questions, related to issues such as species’ behaviors and traits, a species’ spatial distribution, and species’ richness (a count of distinct species) in a particular region: For example, CS data have been used to document the presence and range of rare species and morphs (Borzée et al., 2019; Worthington et al., 2012) and to examine organismal responses to climate change and human activity (Champion et al., 2018; Sanderfoot et al., 2022; Senzaki et al., 2020); digital applications such as eBird and iNaturalist are used to understand species demographics and range expansion (Chardon et al., 2015; Rosenberg et al., 2019); and finally, the availability of this plethora of biodiversity data has facilitated the development of a cost-effective framework for managing wildlife habitats and populations (Aceves-Bueno et al., 2015; Finley et al., 2023; Goldstein et al., 2014; Li et al., 2019).”

Hence, the literature review reveals a tension between the known challenges in CS data and researchers’ ability to employ the data for addressing certain ecological questions. This tension provides the impetus for our study.

Taking a broader perspective, similar issues arise when discussing collective intelligence systems (Malone & Bernstein, 2015; Malone & Klein, 2007). While, of course, ecological CS projects are the primary focus of this work, we view them as a class of broad-participation collective intelligence systems. The literature of collective intelligence suggests that under certain conditions – specifically, when the crowd is diverse in terms of members’ knowledge, experiences, and perspectives, and when individuals’ inputs are largely independent of one another - concerns for participation bias and social influence are largely alleviated, and group members’ dispositions could offset and collectively cancel one another out, yielding an unbiased outcome (Hong & Page, 2004, 2011; Page, 2007; Wolf et al., 2015). Nonetheless, in many circumstances – such as when the project is local – theses conditions are not met, and the community-generated outcome is vulnerable to biases.

Henceforth, an understanding of community members’ preferences and the way in which they aggregate into spatial, temporal and taxonomic biases is of importance.

**R2.2** It would be useful, though, if the paper proposed strategies to mitigate such biases (e.g, design decisions, instructions to contributors).

Response: The comment is well taken. While the focus of this work is and ethnographic investigation of a local CS community, aimed at addressing our primary research questions, and a comprehensive development of bias-mitigation strategies is worthy of much further work, we agreed with the reviewer that it is worthwhile to offer some avenues for future research in that direction, based on our findings. We therefore developed our discussion further, and added proposals for future directions of research and development of bias-mitigation strategies. They are included in the “Practical implications” section, as well as in the Conclusion section.

**R2.3** The project seems to require participants to upload photos, with a reason for not contributing being things like animals appearing fleetingly so that photos could not be captured. Why not allow contributors to report observations using text? A fast moving mammal or bird might not afford a photo opportunity, but contributors certainly could make such reports (thereby mitigating bias!).

Response: The reviewer makes a valid point. Not all monitoring protocols and not all biodiversity monitoring platforms require that an observation includes a photo. iNaturalist, the largest biodiversity monitoring platform uses an evidence-based approach, requesting photos for new observations. Namely, to allow for a community-based quality assurance process, where others could discuss and collectively agree on species identification, it is essential that a photo is provided (in fact, observations that do not include a photo can never progress the quality ladder and can never reach a “Research Grade”).

These, of course, are decisions taken by iNaturalist leaders and apply to all projects. Hence, neither the Tatzpiteva community nor our research team, had a say on this matter.

**R2.4** The paper seems to be hastily written. There are numerous typos (no space to list them here), and the paper uses strange word choices at time (notably, "slant", which seem to be a very informal work choice for a scientific paper. Why not stick to bias?

Response: Following the reviewers’ comment, we dedicated much effort to fixing the language, correcting errors, and generally improving the flow and quality of wiring. We have recruited the services of a professional copy editor, and worked with her on addressing these issues.

Specifically, we now refrain from using informal language. We note that other scientific publications have used “slant” in a similar context (e.g. Greenstein & Zhu, 2012)), but per the reviewers’ request we opted to use an alternative language.

For consistency, when describing individuals’ considerations, we discuss preferences and constraints; and we reserve the term “bias” to when discussing the consequential collective-level phenomenon.

# Bibliography for Rebuttal Letter

Aceves-Bueno, E., Adeleye, A. S., Bradley, D., Tyler Brandt, W., Callery, P., Feraud, M., Garner, K. L., Gentry, R., Huang, Y., & McCullough, I. (2015). Citizen science as an approach for overcoming insufficient monitoring and inadequate stakeholder buy-in in adaptive management: criteria and evidence. *Ecosystems*, *18*, 493-506.

Bernard, H. R. (2017). *Research Methods in Anthropology: Qualitative and Quantitative Approaches*. Rowman & Littlefield Publishers. <https://books.google.com/books?id=2Fk7DwAAQBAJ>

Borzée, A., Baek, H. J., Lee, C. H., Kim, D. Y., Song, J.-Y., Suh, J.-H., Jang, Y., & Min, M.-S. (2019). Scientific publication of georeferenced molecular data as an adequate guide to delimit the range of Korean Hynobius salamanders through citizen science. *Acta herpetologica*, *14*(1), 27-33.

Champion, C., Hobday, A. J., Tracey, S. R., & Pecl, G. T. (2018). Rapid shifts in distribution and high‐latitude persistence of oceanographic habitat revealed using citizen science data from a climate change hotspot. *Global change biology*, *24*(11), 5440-5453.

Chardon, N. I., Cornwell, W. K., Flint, L. E., Flint, A. L., & Ackerly, D. D. (2015). Topographic, latitudinal and climatic distribution of Pinus coulteri: geographic range limits are not at the edge of the climate envelope. *Ecography*, *38*(6), 590-601.

Charmaz, K. (2006). *Constructing Grounded Theory: A Practical Guide Through Qualitative Analysis*. Sage.

Comay, O., Ben Yehuda, O., Benyamini, D., Schwartz‐Tzachor, R., Pe'er, I., Melochna, T., & Pe’er, G. (2020). Analysis of monitoring data where butterflies fly year‐round. *Ecological Applications*, *30*(8), e02196.

Crowston, K., Wei, K., Li, Q., & Howison, J. (2006, 4-7 Jan. 2006). Core and Periphery in Free/Libre and Open Source Software Team Communications. Proceedings of the 39th Annual Hawaii International Conference on System Sciences (HICSS'06),

Emerson, R. M., Fretz, R. I., & Shaw, L. L. (2011). *Writing Ethnographic Fieldnotes* (2nd ed.). University of Chicago Press. <https://books.google.co.il/books?id=k83BlbBHubAC>

Eppler, M. J. (2006). *Managing information quality: Increasing the value of information in knowledge-intensive products and processes*. Springer Science & Business Media.

Finley, D., Dovciak, M., & Dean, J. (2023). A data driven method for prioritizing invasive species to aid policy and management. *Biological invasions*, *25*(7), 2293-2307.

Goldstein, E. A., Lawton, C., Sheehy, E., & Butler, F. (2014). Locating species range frontiers: a cost and efficiency comparison of citizen science and hair-tube survey methods for use in tracking an invasive squirrel. *Wildlife Research*, *41*(1), 64-75.

Greenstein, S., & Zhu, F. (2012). Is Wikipedia Biased? *American Economic Review*, *102*(3), 343-348.

Hong, L., & Page, S. E. (2004). Groups of diverse problem solvers can outperform groups of high-ability problem solvers. *Proceedings of the National Academy of Sciences of the United States of America*, *101*(46), 16385-16389.

Hong, L., & Page, S. E. (2011). Some microfoundations of collective wisdom. In H. Landemore & J. Elster (Eds.), *Collective wisdom: Principles and Mechanisms* (pp. 56-71). Cambridge University Press.

Johnson, R. B., & Christensen, L. (2016). *Educational Research: Quantitative, Qualitative, and Mixed Approaches* (7th ed.). SAGE Publications. <https://books.google.com/books?id=oAFHDQAAQBAJ>

Juran, J. M., Gryna, F. M., & Bingham, R. S. (1979). *Quality control handbook* (Vol. 3). McGraw-Hill New York.

Li, E., Parker, S. S., Pauly, G. B., Randall, J. M., Brown, B. V., & Cohen, B. S. (2019). An urban biodiversity assessment framework that combines an urban habitat classification scheme and citizen science data. *Frontiers in Ecology and Evolution*, *7*, 277.

Malone, T. W., & Bernstein, M. S. (Eds.). (2015). *Handbook of Collective Intelligence*. MIT Press. <https://books.google.com/books?id=JhZuEAAAQBAJ>.

Malone, T. W., & Klein, M. (2007). Harnessing Collective Intelligence to Address Global Climate Change. *Innovations: Technology, Governance, Globalization*, *2*(3), 15-26.

Page, S. E. (2007). *The Difference: How the Power of Diversity Creates Better Groups, Firms, Schools, and Societies*. Princeton University Press. <https://www.nytimes.com/2008/01/08/science/08conv.html>

Rosenberg, K. V., Dokter, A. M., Blancher, P. J., Sauer, J. R., Smith, A. C., Smith, P. A., Stanton, J. C., Panjabi, A., Helft, L., & Parr, M. (2019). Decline of the North American avifauna. *Science*, *366*(6461), 120-124.

Sanderfoot, O., Kaufman, J., & Gardner, B. (2022). Drivers of avian habitat use and detection of backyard birds in the Pacific Northwest during COVID-19 pandemic lockdowns. *Scientific Reports*, *12*(1), 12655.

Senzaki, M., Barber, J. R., Phillips, J. N., Carter, N. H., Cooper, C. B., Ditmer, M. A., Fristrup, K. M., McClure, C. J., Mennitt, D. J., & Tyrrell, L. P. (2020). Sensory pollutants alter bird phenology and fitness across a continent. *Nature*, *587*(7835), 605-609.

Veregin, H. (1999). Data quality parameters. *Geographical information systems*, *1*, 177-189.

Wang, R. Y., & Strong, D. M. (1996). Beyond accuracy: What data quality means to data consumers. *Journal of Management Information Systems*, *12*(4), 5-33.

Wolf, M., Krause, J., Carney, P. A., Bogart, A., & Kurvers, R. H. (2015). Collective intelligence meets medical decision-making: the collective outperforms the best radiologist. *PLoS ONE*, *10*(8), e0134269. <https://doi.org/https://doi.org/10.1371/journal.pone.0134269>

Worthington, J. P., Silvertown, J., Cook, L., Cameron, R., Dodd, M., Greenwood, R. M., McConway, K., & Skelton, P. (2012). Evolution MegaLab: a case study in citizen science methods. *Methods in Ecology and Evolution*, *3*(2), 303-309.
