# Supplementary material for: A local community on a global collective intelligence platform: A case study of individual preferences and collective bias in ecological citizen science
Source: PLoS One. 2024 Aug 26;19(8):e0308552. doi: 10.1371/journal.pone.0308552 (PMC11346665; doi:10.1371/journal.pone.0308552)
Supplement: Attachment — Submitted filename: CitizenScienceBiases - PLOS ONE - RR2 - Rebuttal.docx [file pone.0308552.s006.docx]

**PONE-D-23-35664R1**

**A local community on a global collective intelligence platform: a case study of individual preferences and collective bias in ecological citizen science**

**Reviewer #1**:

The manuscript has consistently improved since last time and I acknowledge the thoroughful revision done by the authors. There are no further comments from my side and I am looking forward to seeing the work published.

**Reviewer #2**: I thank the authors for their revision and the clear response to the reviews in the previous round. I continue to like the paper and have only three remaining substantive comments, both arising from the response/revisions:

Response: We thank reviewer #2 for the insightful comments, which helped us refine and clarify the paper’s contribution. Below we provide detailed responses to the outstanding comments.

1. Framework for Biases: The topic of bias in citizen-generated data is important. A recent article proposed a framework for understanding "socio-ecological biases" in citizen science data (Carlen et al., 2024). Given the apparent relevance to your work, it might help to relate your perspective to their framework. I don't think they have "scooped" you, but on a first glance, your in-depth qualitative work can provide support for aspects of their framework and perhaps challenge others.

Response: We appreciate the comment. Indeed, Carlen et al.’s 2024 paper, which addresses observer-based biases is relevant to our work, and we have referred to their work in multiple places throughout the paper in the previous revision as well (specifically in the introduction, related work, discussion, and conclusion). But we accept your suggestion to further relate to their paper in the discussion part.

Indeed, as you noted, the findings from our empirical study lend support to certain aspects of their framework, and at the same time challenges other assertions. And then, some other aspects of their work, specifically – the participation filter – are out of the scope of our inquiry, since they discuss social factors that lead to the inclusion/exclusion of people from the community of observers, which we did not study at all in this case.

Following your suggestion, we slightly extended our discussion on p. 22, and mentioned where the main factors that emerged from our inquiry correspond to their framework (Specifically, our *Recordability* and *Convenience* categories correspond to a large extent to Carlen et al.’s *Detectability* and *Sampling* filters, respectively).

We further highlighted (on p. 22) an important point where we diverge: Carlen et al.’s conceptual framework depicts the monitoring process as comprised of (or, subject to) a series of “filters”, where each filter places a restriction on the possibility of reporting observed species, gradually moving away from the true state of nature. Our findings challenge the “linearity” of this model, or the assumption that these filters are independent and sequentially applied; we offer that in practice the considerations are often intertwined.

Finally, on p. 30, as part of the “Limitations and future research directions” section, we now turn readers’ attention to the importance of participation bias, citing Carlen’s important work.

2. Data Quality: As noted on page 4, traditional approaches to data quality focus on fitness-for-use and, to assess this, "we must consider the possible uses." However, it may be that uses are not be fully known at the time a project is designed and launched and/or may change (consider, e.g., Pharr et al., 2023, where data from a CS project is combined with US government light and noise data to answer questions not considered when the original CS project was designed). As you note, "CS data can be valuable for addressing a variety of research questions." In that context, there has been a suggestion that data quality in citizen science (and crowdsourcing more broadly) can sometimes be considered from a "use-agnostic" perspective (Wiersma et al., 2024; Lukyanenko et al., 2014). In that context, it is unclear how contributors' preferences (versus the goals of project sponsors) affect the nature of bias and resulting impacts on data quality. For example, project sponsors likely have specific goals (e.g., understanding the distribution and prevalence of (focal) species), whereas contributors may produce biases along the dimensions identified in your analysis. Some discussion of the inherent tension between viewing data quality from a fitness-for-use perspective and the way in which data can become biased as a results of that focus imposed by project goals (in addition to, and perhaps in different ways from, the biases you discuss in the paper based on behavior and preferences of contributors) would deepen the analysis of the relationship between bias and data quality.

Response. The comment is well taken. The definition of data quality as “fitness for use” is commonly accepted, yet, as R2 suggests, often when collecting data, we cannot consider all possible future uses. Hence, we accept the “use-agnostic” perspective, and our analysis attempts to provide a broad assessment of biases that is independent of any particular research questions. Following R2’s comment, we now explain this in the Introduction (p. 4) of the revised manuscript and revisit the issue in the Discussion section. We have added a new paragraph to discuss this point (under “Limitations and future research directions”).

3. Platform-design-induced bias: The only response to my previous comments that was less that satisfying was the one regarding the photo requirement. I realize this is iNaturalist-driven, rather than Tatzpiteva-driven, but it seems clear that such a requirement is a big source of systematic bias. One of your key findings is that "recordability" drives the decision to (not) report, or in the words of one of your respondents "the ability to take a picture." Unlike issues related to personal preferences or convenience, this seems an artifact of underlying decisions of the platform. While a photo might be useful in enabling verification, it seems a mechanism to induce severe bias in certain contexts (e.g., low light, fast-moving animals) by preventing participants from reporting observations they might otherwise be inclined to report. As Lukyanenko et al. (2019) show, it is possible to perform post hoc processing on textual data to get a high level of classification performance on textual data (no photos) that lacks species identification.

Response. In our analysis, we include such factors under “Constraints”, and this is why we speak of considerations that include both preferences and constraints (see also, Arazy & Malkinson, 2021). Specifically, the ability to take a photo constrains observers’ reporting ability. Yes, this is a constraint that is imposed by the platform (or the project’s sponsors), and different platforms impose different constraints.

Following your comment, we have now extended our discussion of this point. In the revised Discussion section, under the sub-section “Contribution to the literature on biases in ecological citizen science”, we highlight the unique characteristics of evidence-based CS projects (in contrast to expertise-based projects). Additionally, we included a new paragraph (under “Limitations and future research directions”) to discuss the implications of the requirement to include a photo.

In conclusion, this paper stands to make a strong contribution to our understanding of preferences and biases among contributors to citizen science projects. The comments above are intended in the spirit of better refining the message and contextualizing it with respect to related work.

Response. Thank you for re-stating the paper’s contribution. We appreciate your thoughtful comments, and we have carefully worked to address them, as described above. We feel that they have indeed helped us to better convey our paper’s contribution and contextualize our discussion with reference to prior works in the area.

References

Carlen, Elizabeth J, Cesar O Estien, Tal Caspi, Deja Perkins, Benjamin R Goldstein, Samantha ES Kreling, Yasmine Hentati, Tyus D Williams, Lauren A Stanton, Simone Des Roches, Rebecca F Johnson, Alison N Young, Caren B Cooper, Christopher J Schell (2024). A framework for contextualizing social-ecological biases in contributory science. People and Nature, 6(2), 377-390.

Lukyanenko, Roman, Jeffrey Parsons, Yolanda Wiersma, Mahed Madadah (2019). Expecting the unexpected: effects of data collection design choices on the quality of crowdsourced user-generated content. MIS Quarterly, 43(2), 623-647.

Pharr, Lauren D, Caren B Cooper, Brian Evans, Christopher E Moorman, Margaret A Voss, Jelena Vukomanovic, Peter P Marra (2023). Using citizen science data to investigate annual survival rates of resident birds in relation to noise and light pollution. Urban Ecosystems 26(6), 1629-1637.

Wiersma, Yolanda F, Tom Clenche, Mardon Erbland, Gisela Wachinger, Roman Lukyanenko, Jeffrey Parsons (2023). Advantages and Drawbacks of Open-Ended, Use-Agnostic Citizen Science Data Collection: A Case Study. Citizen Science: Theory and Practice, 9(1), 5pp.
